# The contribution of cattle urine and dung to nitrous oxide emissions: Quantification of country specific emission factors and implications for national inventories

**DOI:** 10.1016/j.scitotenv.2018.04.152

**Published:** 2018-09-01

**Authors:** D.R. Chadwick, L.M. Cardenas, M.S. Dhanoa, N. Donovan, T. Misselbrook, J.R. Williams, R.E. Thorman, K.L. McGeough, C.J. Watson, M. Bell, S.G. Anthony, R.M. Rees

**Affiliations:** a School of Environment, Natural Resources and Geography, Bangor University, Bangor LL57 2UW, UK; b Rothamsted Research, North Wyke, Devon EX20 2SB, UK; c ADAS Boxworth, Battlegate Rd., Cambridge CB23 4NN, UK; d Agri-Food and Biosciences Institute, 18a, Newforge Lane, BT9 5PX, Belfast, UK; e Scotland's Rural College (SRUC), West Mains Road, Edinburgh EH9 3JG, UK; f ADAS Wolverhampton, Titan 1 offices, Coxwell Avenue, Wolverhampton Science Park, Wolverhampton WV10 9RT, UK

**Keywords:** Grassland, Greenhouse gas, Nitrous oxide, Cattle, Urine patch, Dung pat, Nitrification inhibitor, Dicyandiamide, Inventory

## Abstract

Urine patches and dung pats from grazing livestock create hotspots for production and emission of the greenhouse gas, nitrous oxide (N_2_O), and represent a large proportion of total N_2_O emissions in many national agricultural greenhouse gas inventories. As such, there is much interest in developing country specific N_2_O emission factors (EFs) for excretal nitrogen (EF_3,_ pasture, range and paddock) deposited during gazing. The aims of this study were to generate separate N_2_O emissions data for cattle derived urine and dung, to provide an evidence base for the generation of a country specific EF for the UK from this nitrogen source. The experiments were also designed to determine the effects of site and timing of application on emissions, and the efficacy of the nitrification inhibitor, dicyandiamide (DCD) on N_2_O losses. This co-ordinated set of 15 plot-scale, year-long field experiments using static chambers was conducted at five grassland sites, typical of the soil and climatic zones of grazed grassland in the UK. We show that the average urine and dung N_2_O EFs were 0.69% and 0.19%, respectively, resulting in a combined excretal N_2_O EF (EF_3_), of 0.49%, which is <25% of the IPCC default EF_3_ for excretal returns from grazing cattle. Regression analysis suggests that urine N_2_O EFs were controlled more by composition than was the case for dung, whilst dung N_2_O EFs were more related to soil and environmental factors. The urine N_2_O EF was significantly greater from the site in SW England, and significantly greater from the early grazing season urine application than later applications. Dycandiamide reduced the N_2_O EF from urine patches by an average of 46%. The significantly lower excretal EF_3_ than the IPCC default has implications for the UK's national inventory and for subsequent carbon footprinting of UK ruminant livestock products.

## Highlights

• First co-ordinated experiments in UK to generate data for country specific grazing excretal N_2_O EF
• Urine had a significantly greater average N_2_O EF (0.69%) than dung (0.19%).
• The combined excretal N_2_O EF was 0.49%, <25% of the IPCC default value for cattle.
• DCD reduced the N_2_O EF from urine patches by an average of 46%.
• Urine N_2_O was controlled by its composition, dung N_2_O was related to soil and environmental factors.

## Graphical abstract

**Figure:**
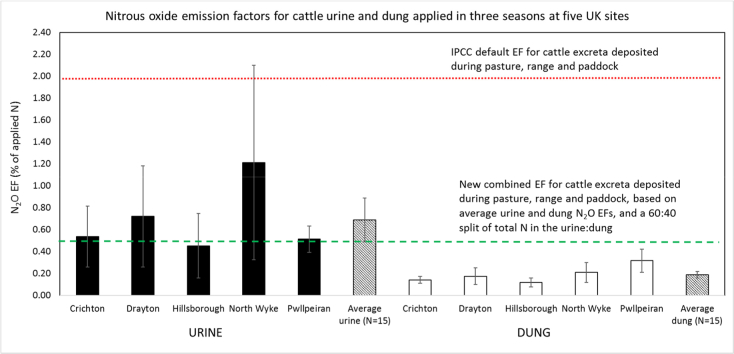

## 1 Introduction

Grazed grasslands support a significant proportion of sheep and cattle production throughout Europe and other parts of the World, converting human-inedible plant biomass into human edible animal products but with generally low nitrogen (N) use efficiencies. The ruminant animal converts much of the organic N in plant biomass into highly reactive and bioavailable N (Nr), particularly as excreted in the urine. It is thought that 3.08 Mt. of N is deposited by grazing livestock in Europe, and this value is thought to be as much as ca. 0.61 Mt. N in the UK (UNFCCC, 2016). It is well documented that urine additions to grassland soils result in significant quantities of N_2_O production and emission, mainly due to the soil microbial processes of nitrification and denitrification (Selbie et al., 2015), following the addition of readily available N and carbon (C), and the effects of significantly increased percentage of water-filled pore space (WFPS) within the urine patch (van der Weerden et al., 2017).

Deposition of N in urine patches can represent an equivalent application rate of 200–2000 kg N ha^−1^ (Selbie et al., 2015), depending on the protein content of the sward, livestock type, age and stage of lactation. A meta-analysis by Selbie et al. (2015) indicates average urine patch N loading rates of 613 kg N ha^−1^ and 345 kg N ha^−1^ for dairy cows and beef cattle, respectively. Clearly, N loading rates in urine patches are in excess of optimal plant use efficiency, increasing the risk of excess N being lost to the environment via nitrate (NO_3_^−^) leaching (de Klein and Ledgard, 2001; Di and Cameron, 2007), ammonia (NH_3_) volatilization (Lockyer and Whitehead, 1990; Laubach et al., 2013; Burchill et al., 2017), N_2_O (Di and Cameron, 2008; Krol et al., 2016; Van der Weerden et al., 2017; Minet et al., 2018) and N_2_ (Clough et al., 1998) emissions. All of these N loss pathways (except N_2_O losses) typically represent a significant agronomic loss, and all but N_2_ loss have detrimental effects on the environment.

At these high rates of N loading, the N_2_O emission is likely to be disproportionally greater than emissions from N sources applied at lower N loading rates, e.g. typical fertiliser N applications at agronomic rates. A curvilinear response of N_2_O emissions to N loading has been shown previously, e.g. Cardenas et al. (2010) for fertiliser N (NH_4_NO_3_) applications between 0 and 375 kg N ha^−1^ to grazed swards. Bell et al. (2015) also showed a non-linear response of N_2_O fluxes to NH_4_NO_3_ applications (0–400 kg N ha^−1^) to cut grass. More specifically for urine applications, de Klein et al. (2014) demonstrated greater N_2_O emissions, as a percentage of N applied, i.e. emission factors (EFs), (0.34%) from urine patches receiving an N loading of 1200 kg ha^−1^ compared to urine patches with a lower N loading (0.10% from a loading of 200 kg ha^−1^) on a freely draining soil, although a linear relationship between N_2_O EFs and urine N loading was observed on a poorly drained soil. van Groenigen et al. (2005) found no effect of N loading in urine patches on the N_2_O EF.

**Fig. 1:**
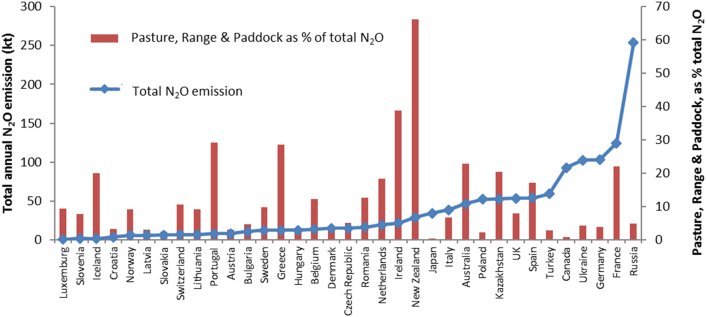
Annual (year 2014) agricultural N_2_O emissions and direct N_2_O emissions from excreta deposited by grazing livestock (pasture, range and paddock), expressed as a percentage of the total agricultural N_2_O emission, from different nations (source: UNFCCC, 2016).

For excretion during cattle grazing, the default IPCC N_2_O EF (pasture, range and paddock) is 2% for (combined excretal urine + dung EF) (cf to 1% for fertiliser N), whilst the N_2_O EF for sheep excretal N during grazing is only 1% (IPCC, 2006). UNFCCC submissions for 2015 from different countries (using IPCC Tier 1/2, 2006 Guidelines) show that direct N_2_O emissions following N deposited to soil by grazing livestock represents from <5% (e.g. in Japan) to >65% (in New Zealand) of total national direct soil N_2_O emissions (Fig. 1), with greater contributions coming from countries where livestock graze for significant periods of the year (UNFCCC, 2016). As this source of direct N_2_O emissions is significant to many national agricultural greenhouse gas inventories, there is increasing interest in developing country specific EFs that better reflect national soils and climatic conditions (e.g. Krol et al., 2016 for Ireland).

Most Nr excreted during grazing is in the urine, which is mostly comprised of urea that requires hydrolysis to free NH_4_^+^ (Selbie et al., 2015). In dung, most N is in the organic form, and requires mineralisation over a longer time period to provide a pool of NH_4_^+^ for nitrification and NO_3_^−^ for denitrification. The split between urine and dung for total excretal N will depend on dietary protein intake compared with requirement by the animal (as protein intake increases above requirement proportionally more N will be excreted as urine (Broderick, 2003; Reed et al., 2015), and partially on the digestibility of the protein in the diet (with a higher proportion of less digestible protein being excreted as faecal N). The UK GHG and ammonia emission inventories to date have assumed 60% of total N excretion by cattle to be as urine and 40% as dung (Webb and Misselbrook, 2004), in common with other Western European countries (Reidy et al., 2008). Disaggregating emissions to urine and dung offers an improved understanding of the sources of N_2_O from grazed pastures, and hence how they could be mitigated.

Since direct N_2_O emissions from grazing livestock represent such a large term in national agricultural greenhouse gas inventories, there has been significant interest in understanding factors that contribute to N_2_O production and emission from this source, e.g. soil type (Clough et al., 1998), urine composition (Kool et al., 2006; Gardiner et al., 2016), weather conditions (Krol et al., 2016), and in exploring strategies to reduce emissions. For example, Monaghan and de Klein (2014) have suggested restricting the duration of autumn and winter grazing to reduce higher N_2_O fluxes associated with urine deposition to wet soils (Qui et al., 2010; Krol et al., 2016). Other studies have explored how manipulating the natural urine composition, e.g. hippuric acid content, can reduce N_2_O production from the urine patch (Clough et al., 2009), and there has been much interest in the use of synthetic nitrification inhibitors to reduce both NO_3_^−^ leaching and N_2_O emissions from urine patches (Hatch et al., 2005; Di and Cameron, 2012; Barneze et al., 2015). New Zealand and Irish research groups have taken this a step further, in exploring how the nitrification inhibitor dycandiamide (DCD) can be delivered to urine patches to reduce N_2_O emissions, e.g. through boluses (Ledgard et al., 2008), in drinking water (Welten et al., 2014), and in feed (Luo et al., 2015; Minet et al., 2016, Minet et al., 2018). However, recent publicity and research has demonstrated that there are potential unintended consequences of using nitrification inhibitors, such as contamination of milk products, e.g. via root or foliar uptake (Marsden et al., 2015; Pal et al., 2016) and increased ammonia emissions (Lam et al., 2017), so researchers are exploring new inhibitor products, including biological nitrification inhibitory compounds targeted at ruminant production (Gardiner et al., 2016; Balvert et al., 2017; Luo et al., 2018) that may be deemed more acceptable to the public in the future.

The UK greenhouse gas R&D community undertook a large number of field trials to quantify N_2_O EFs from a range of different N sources (viz, different fertiliser N forms, different manure types, and urine and dung deposited by grazing livestock (Chadwick et al., 2011), as part of a larger programme to improve the reporting tool for the national inventory of agricultural greenhouse gas emissions that better represents the soils, climate and N management in the UK. In this paper, we summarise the results of the first co-ordinated set of plot-based experiments aimed at generating new N_2_O emissions data for disaggregated urine and dung deposition to soil, from which country specific N_2_O EFs can be derived that are relevant to UK soils and climate. Some of the individual site experimental results can be found in Bell et al. (2015) and Cardenas et al. (2016). In the experiments, we tested whether season of urine and dung deposition (early grazing, mid grazing, later grazing period) influenced the N_2_O EF. We also tested the efficacy of the nitrification inhibitor, dicyandiamide (DCD), to reduce N_2_O emissions. An additional reference treatment was included in each experiment, a standardised artificial (synthetic, produced in the laboratory) urine treatment, with the aim of using the information from this treatment to help disentangle the effects of urine composition from soil and climate effects on N_2_O EFs.

The specific aims of this study were to: i) determine separate direct N_2_O EFs for cattle urine and dung, ii) determine if season of urine and dung deposition affected the direct N_2_O emission, iii) assess the effects of site on direct N_2_O emissions from urine, iv) evaluate the efficacy of the nitrification inhibitor, DCD, to reduce direct N_2_O emissions from urine, and v) assess the influence of using the combined experimentally derived urine and dung N_2_O EF on national N_2_O emissions.

## 2 Materials and methods

### 2.1 Site selection

**Fig. 2:**
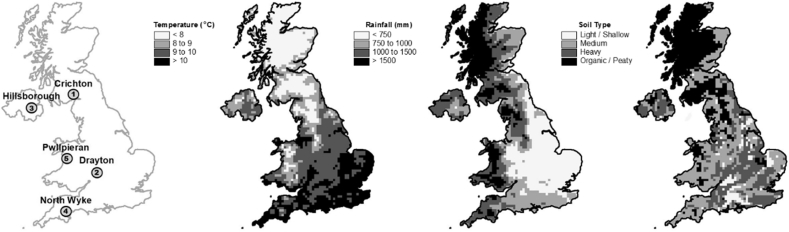
Site location, climate average (1981 to 2010) rainfall and temperature, and distribution of dominant soil types.

Five experimental sites were selected to cover the range of typical grassland soils and climate throughout the UK, with two sites in England, one in Scotland, one in Wales and one in Northern Ireland (see locations in Fig. 2). Descriptions of the sites are shown in Table 1. There have been few previous studies in the UK where N_2_O EFs have been quantified from urine and dung deposition that are IPCC compliant (IPCC, 2000, IPCC, 2006) (i.e. where emission measurements were also made from control plots, and where measurements lasted for up to 365 days), that these sites needed to provide an appropriate range of soil texture and climate. However, some practicality was also considered in site selection; location could not be excessively far from a research base to ensure timely measurements, since >30 measurement occasions were needed during each 12-month experimental period. Four measurement teams, from different UK organisations, ADAS, AFBI, Rothamsted Research - North Wyke and SRUC, conducted the 15 experiments, following an agreed joint experimental protocol to ensure aspects of the urine and dung management, chamber deployment, and ancillary measurements were made in a similar way.

**Table 1:** Site and soil characteristics. Soil parameters for the 0–10 cm layer.

Site	Country	Altitude (m)	30 yr average annual rainfall (mm)	30 yr average annual air temperature (°C)	Clay content (%)	Soil pH	Organic matter content (%)	Bulk density (g cm^−3^)
Crichton	Scotland	50	1140	9.1	13	5.6	3.05	1.07
Drayton	England	47	628	10.3	59	7.6	4.84	0.90
Hillsborough	Northern Ireland	128	908	9.0	23	6.0	9.82	0.90
North Wyke	England	185	1042	10.0	37	5.7	5.40	0.62
Pwllpeiran	Wales	213	1570	10.0	29	5.5	5.40	0.92

Experiments were conducted on established grasslands where the dominant pasture plant was *Lolium perenne*, which is typical of UK livestock systems (Fig. 2). Each experiment comprised three replicate blocks with five treatments, so a total of 15 plots were sampled on every occasion. There were 5 urine patches or 5 dung pats per plot (to account for variability in soil conditions) with one chamber per patch/pat, hence 45 chambers per experiment. There were also control plots that received no treatment application. Applications were made in the spring, summer and autumn (to separate plots), to simulate excretal deposition in early-, mid- and late- grazing season. Livestock were excluded from grazing the experimental areas at least 6 months prior to the start of any experiment. This minimised any direct effect of previous deposition of excreta on N_2_O emissions.

### 2.2 Urine and dung provision

The experimental design resulted in the need for ca. 200 l of fresh cattle urine and ca. 300 kg dung for each experiment. Urine and dung were collected from the institutions summarised in Table 2 within 7 days of an experiment starting, and stored in sealed containers (un-acidified) at <4 °C. Table 2 summarises the origin of the urine and dung used in each experiment.

**Table 2:** Sources of urine and dung for the experiments.

	Cattle type	Age and approx. live weight	Diet
Crichton	Lactating dairy cows	3–7 years old (ca 600 kg)	Grass silage + concentrates (6.5 kg DM head^−1^ day^−1^)
Drayton	Lactating dairy cows	6 years old (ca. 600 kg)	Concentrate blend, hay, straw, grass silage, maize silage
Hillsborough	Lactating dairy cows	3–5 years old (ca. 600 kg)	Grass silage + concentrates (4 kg DM head^−1^ day^−1^)
North Wyke	Lactating dairy cows	6 years old (ca. 600 kg)	Concentrate blend, hay, straw, grass silage, maize silage
Pwllpeiran	Lactating dairy cows	6 years old (ca. 600 kg)	Concentrate blend, hay, straw, grass silage, maize silage

### 2.3 Treatments

Urine and dung were removed from cold storage at least 12 h before application to the soil, to allow them to attain ambient temperature prior to application to the soil. Urine and dung were applied at typical N loading rates and volumes. The volumetric loading rate was based on a typical 1.8 l per urination event (Misselbrook et al., 2016). Since the N content of the collected urine varied between feeding trials, the N loading rate varied between an equivalent rate of 340 and 570 kg ha^−1^, with an average loading rate of 455 kg N ha^−1^ (see Table 4a). Dung was applied at an equivalent rate of 20 kg m^−2^, representing typical deposition by grazing cattle (Sugimoto and Ball, 1989), with an average loading rate of 835 kg N ha^−1^ (range 625–1020 kg N ha^−1^; Table 4b). Since urine composition could not be controlled between experiments, a standard artificial urine treatment was included at each site as a reference treatment. This was to allow the effects of soil and climate to be determined. The artificial urine recipe of Kool et al. (2006) was used in all experiments.

A urine treatment containing DCD was added, with DCD applied at a rate of 10 kg ha^−1^ equivalent (supplying 6.5 kg N ha^−1^ equivalent), and was mixed with urine (only) just before application, to maximise initial co-location of DCD and NH_4_^+^ in the soil profile. This approach also simulated the effect of delivering DCD via boluses (Ledgard et al., 2008), feed (Luo et al., 2015; Minet et al., 2016, Minet et al., 2018) and via water troughs (Welten et al., 2014). The following treatments were established:

• Urine (target 500 kg N ha^−1^)
• Urine + DCD (target 500 kg N ha^−1^ + 6.5 kg N ha^−1^ in DCD)
• Artificial urine (500 kg N ha^−1^; Kool et al., 2006 recipe)
• Dung (target 800 kg N ha^−1^)
• Control (no additions)

Five chambers were set up for each treatment plot, and three replicate plots per treatment were arranged in three blocks. Table 4a, Table 4b shows application rates for urine and dung at each site.

### 2.4 Treatment applications

Urine treatments were applied to an area of 0.6 m × 0.6 m within a frame to facilitate infiltration (rather than runoff) using a watering can. After application, static chambers were inserted centrally into this area. Dung pats were spread to cover the entire area within the chamber. We recognise that urine and dung patches are not normally this large, and have “edges”, but this method of application was deemed the most appropriate to simulate the urine patch and dung pat. It is possible that by applying the N source across the whole area of the chamber that N_2_O production and emission may have been affected, but there is no evidence to suggest that this would result in either an under- or over-estimate of the true emission (Marsden et al., 2016). In addition to the urine and dung patches that were established for the N_2_O chamber measurements, larger areas of grassland (2 m × 2 m) on each plot (i.e. three replicates per treatment) were treated with either urine or dung at the same rate, allowing multiple soil sampling occasions for soil NO_3_^−^, soil NH_4_^+^ and soil moisture.

### 2.5 Nitrous oxide measurements

We used the non-steady state static chamber approach to measure N_2_O fluxes (Cardenas et al., 2016). The shape and size of the chambers were 0.4 m × 0.4 m × 0.25 m (high) for the ADAS, North Wyke and AFBI experiments, and 0.4 m diameter × 0.3 m (high) for the SRUC experiments, with individual chamber areas of 0.16 and 0.13 m^2^, respectively. Chambers were opaque. Chamber headspace sampling followed the protocol detailed in Chadwick et al. (2014), whereby chambers were closed for a period of 40 min and a headspace sample taken at this time (T_40_). Ten ambient air samples (5 at the start and 5 at the end of the chamber closure period) were used to provide the T_0_ concentration. Gas samples were placed in pre-evacuated 20 ml vials and transported back to individual laboratories for analysis by gas chromatography. Five chambers were assigned randomly per plot; these generated one mean flux per plot. The headspace sampling assumed a linear increase in headspace N_2_O concentration (as evidenced by previous research; Chadwick et al., 2014). This linear response was checked on each sampling occasion by measuring the headspace concentration at 10 min intervals up to 60 min after closure, from one chamber per block.

Sampling frequency was 4–5 times in the first week after treatment application, 4–5 times in the second week, 2 times per week for the next two weeks, then once per week for 1 month. Sampling frequency was then reduced further, eventually to once per month until the end of the experiment (12 months), resulting in ca. 30 samples over the 12-month period following application in order to comply with IPCC recommendations (IPCC, 1996).

### 2.6 Other measurements

#### 2.6.1 Dung and urine composition

Dung and urine sub-samples were taken on the day of application and characterised by measuring pH (in H_2_O), dry matter (DM), total N (by Kjeldahl) and total organic carbon content, either using a modified Walkley-Black approach, or analysis by a TOC analyser (uv persulphate oxidation). The readily available N content was also determined, i.e. ammonium N (NH_4_^+^-N) and nitrate N (NO_3_^−^-N). In addition, two 30 ml sub-samples of urine were taken from each block and preserved by diluting 1:3 with HPLC grade deionised water. The first sample was acidified by adding 1 M H_2_SO_4_ to reduce the pH to 3 (using a pH meter). To the second sample, 100 μl chloroform was added. Both sub-samples were stored at −20 °C before analysis for urea, hippuric acid, allantoin, uric acid and creatinine, by HPLC (using methods described in Kool et al., 2006).

#### 2.6.2 Soil mineral N and moisture determination

Soil NH_4_^+^-N and NO_3_^−^-N: Soil samples (0–10 cm) were taken from the dedicated sampling areas of each plot on 10–12 occasions during the 12-month experiment. Fresh soil was passed through a 5 mm sieve before extracting with 2 M KCl and filtering. Filtrates were frozen prior to analysis for NH_4_^+^-N and NO_3_^−^-N concentrations by colorimetric determination (Singh et al., 2011) using Skalar segmented flow analysers.

Soil moisture content: sub-samples of the sieved soil were weighed (fresh weight) before oven drying at 105 °C overnight, and then reweighed. Soil moisture content was converted to %WFPS using the bulk density of the site (see below) and a particle size density of 2.65 g cm^3^.

#### 2.6.3 Bulk density

Three representative bulk density measurements were made per site, one per block (walking and sampling a ‘W’ route across each block), at the start of the experiment, using 100 cm^3^ bulk density rings, and drying at 105 °C overnight.

#### 2.6.4 Weather data

Daily rainfall and hourly air and soil (0–5 cm) temperature were recorded on site, or daily data used from a nearby weather station (within 1 km) (Table 3).

**Table 3:** Weather and soil data for the different urine and dung applications. Archived data sources: Bell et al. (2017); Cardenas et al. (2017); McGeough et al. (2017); Thorman et al., 2017a, Thorman et al., 2017b.

			Whole measurement period		Initial 30 d after application			Day of application		
Site	Grazing season period	Measurement period	Total rainfall (mm)	Average temperature (°C)	Total rainfall (mm)	Average temperature (°C)	Average WFPS (%)	Daily rainfall (mm)	Average daily temperature (°C)	WFPS (%)
Crichton	Early	03/04/12–18/03/13	1325	8.6	60	7.5	51.9	0.1	5.3	41.4
	Mid	27/06/12–10/06/13	1262	8.7	125	14.7	58.3	2.3	12.9	54.8
	Late	08/10/12–25/09/13	1142	9.1	137	7.6	69.8	0.0	8.4	64.6
Drayton	Early	02/05/13–17/04/14	726	10.7	78	10.9	40.1	0.0	10.0	43.6
	Mid	15/08/13–29/07/14	769	9.4	50	15.8	31.0	1.4	20.5	27.5
	Late	17/10/13–14/10/14	787	8.0	123	9.0	45.2	0.0	12.5	42.5
Hillsborough	Early	2/04/12–1/04/13	1191	8.0	77	6.3	84.1	1.4	8.3	88.5
	Mid	25/06/12–24/06/13	1110	8.2	102	14.0	89.3	0.0	12.1	83.1
	Late	17/09/12–16/09/13	1080	8.2	135	8.8	89.9	0.8	10.9	89.7
North Wyke	Early	15/05/12–09/05/13	1405	9.6	143	13.9	57.5	0.6	7.4	58.3
	Mid	03/07/12–11/06/13	1246	9.4	103	13.6	62.0	7.6	15.4	62.4
	Late	26/09/12–10/09/13	1288	9.5	160	9.1	63.1	11.1	12.4	71.6
Pwllpeiran	Early	11/04/13–27/03/14	1878	10.4	96	8.4	44.8	5.7	7.8	44.1
	Mid	04/07/13–16/06/14	1844	10.8	61	17.2	36.7	0.0	14.4	44.1
	Late	12/09/13–27/08/14	1841	10.6	98	13.6	44.8	13.6	14.4	42.2

### 2.7 Data processing and statistics

The N_2_O flux for each chamber was calculated by entering data for the sample vials N_2_O concentration, air temperature, closure period and chamber heights into a standard spreadsheet used by all project partners. The mean of the 5 chambers per plot was calculated and used for subsequent calculations of cumulative emissions, using the trapezoidal rule (Cardenas et al., 2010). EFs were calculated by subtracting cumulative N_2_O emissions from control plots from treatment plots in the same block. For the urine treatment with DCD the N content in the DCD was taken into account for the calculation of the EF. EFs uniformity of distribution were checked and, if necessary, Box Cox transformation was used on all N_2_O data to normalise distribution. Statistical analyses were designed to test:

i) the effect of geographical site on N_2_O EFs for the different treatments
ii) the effect of season of application on N_2_O EFs for different treatments
iii) the difference between urine and dung N_2_O EFs
iv) the effect of DCD in reducing N_2_O EFs from urine application

Treatment effects and their interactions were evaluated using the F-test in analysis of variance (ANOVA) of each site according to the randomised block design. Multiple comparison of treatment means, if significant, were tested using the Tukey method (Hsu, 1996). When ‘treatment × season’ interaction was significant then treatments were compared within each season, and seasons were compared with each treatment. In addition, all five sites were combined using REML Meta-analysis in Genstat (VSN International, 2015) where the fixed effects model included main effects and interactions of sites, treatments and seasons (random effects model accounted for the design factors).

Multiple regression analysis (forward selection procedure in Genstat) was used to explore the key soil (% clay, pH, initial % WFPS, average WFPS for first 30 days), environment (average temperature for the first 30 days, average temperature for 365 days after application, total rainfall for the first 30 days, total rainfall for 365 days after application) and urine/dung composition (total urine/dung N content, total urine urea content, total urine/dung ammonium content, uric acid content, hippuric acid content, allantoin content, creatinine content, N application rate) factors that controlled the cumulative N_2_O fluxes and N_2_O EFs. The main effects of up to (maximum) 10 terms was estimated. No interaction terms were included for selection. In developing a multiple regression model, correlation among the predictor factors (known as multicollinearity) can affect model equation stability. For this modelling exercise, we used the statistical package Genstat (Genstat 18th Ed.; VSN International, 2015), which has the built-in facility to check for any multicollinearity issues (any such problem can be dealt with by using Genstat Procedure “Ridge” regression which incorporates Principal Component (PCA) regression).

## 3 Results

### 3.1 Urine and dung composition

The N content of the urine used in the 15 experiments (Table 4a) were typical for cattle urine (Dijkstra et al., 2013; Selbie et al., 2015; Gardiner et al., 2016), ranging from 6.8 to 11.4 g l^−1^ (average 9.11 g l^−1^ ± 0.35). In most cases urea-N represented between 60 and 100% of the total N content. However, for the three experiments at Hillsborough, the low urea-N content of the urine was linked to a high urine ammonium-N content (Table 4a), indicating hydrolysis of urea prior to application to the soil. Since urea hydrolysis is such a rapid process once urine has been deposited on the soil, we do not consider the N_2_O emissions from the three Hillsborough experiments to have been directly affected by this.

**Table 4a:** Average urine composition, and N and C loading rates for each experiment. For urine + DCD treatments, an additional 6.5 kg N ha^−1^ was supplied in the inhibitor. DM = dry matter. Archived data sources: Bell et al. (2017); Cardenas et al. (2017); McGeough et al. (2017); Thorman et al., 2017a, Thorman et al., 2017b.

		Urine							Artificial urine						
Site	Grazing season period	Total N loading (kg ha^−1^)	pH	DM (%)	Total N (g l^−1^)	Urea-N (mg l^−1^)	NH_4_^+^-N (mg l^−1^)	NO_3_^−^-N (mg l^−1^)	Total N loading (kg ha^−1^)	pH	DM (%)	Total N (g l^−1^)	Urea-N (mg l^−1^)	NH_4_^+^-N (mg l^−1^)	NO_3_^−^-N (mg l^−1^)
Crichton	Early	480	–	4.9	9.60	6332	120	–	180	–	1.4	3.60	1318	–	–
	Mid	420	–	4.6	8.40	8127	240	–	425	–	3.5	8.50	9264	–	–
	Late	435	–	4.9	8.70	6231	100	–	425	–	3.5	8.50	9264	–	–
Drayton	Early	540	7.5	5.5	10.80	10,780	825	0.5	501	7.1	4.6	10.01	9820	25	0.5
	Mid	454	9.0	4.5	9.07	8540	4870	1.5	495	7.3	3.9	9.91	8340	39	0.0
	Late	471	8.1	5.2	9.43	8480	315	0.0	495	7.1	4.7	9.90	10,040	25	0.0
Hillsborough	Early	432	9.0	5.5	8.64	2900	6917	0.0	510	7.7	4.6	10.20	7335	100	115.0
	Mid	338	8.9	5.3	6.75	375	5862	27.0	502	7.6	5.0	10.04	8035	55	126.0
	Late	354	9.0	4.2	7.07	767	6216	41.0	504	8.2	4.5	10.08	8048	88	163.0
North Wyke	Early	405	8.3	5.3	8.10	6521	554	0.0	440	8.2	4.3	8.80	7079	18	0.1
	Mid	429	7.3	4.8	8.57	6284	1230	1.0	481	7.5	4.2	9.61	6833	<50	0.4
	Late	435	9.2	4.5	8.70	7382	2020	2.5	423	7.4	3.4	8.45	7774	<50	0.8
Pwllpeiran	Early	565	9.3	5.6	11.30	10,100	2743	0.3	495	9.3	4.4	9.91	9620	315	0.3
	Mid	568	7.8	5.5	11.37	6840	822	0.3	498	7.4	3.9	9.96	7840	25	0.2
	Late	505	7.8	4.7	10.10	8820	115	0.3	508	7.5	4.1	10.15	10,040	25	0.0

Concentrations of the purine derivatives in the urine varied markedly between the different seasons of collection for the different experiments at each site, and between sites (Table 5).

This reflects differences in the diets that cattle were fed prior to collection of the urine on each occasion (see Table 2 for a summary of the diets), and differences between cattle groups at each collection site. However, concentrations are typical of those reported in the literature (Dijkstra et al., 2013; Selbie et al., 2015; Gardiner et al., 2016). The measured N contained in the purine derivatives represented from 3 to 28% of the total N content of the urine (average 12.5% ± 0.02).

The total N content of the dung ranged from 3.4 to 48.0 g kg^−1^ (DM), whilst the DM content ranged from 10.6–36.2% (Table 4b). The total N loadings in the urine and dung treatments were typical for cattle, 338–568 kg ha^−1^ (average 455 ± 17.6) and 625–1020 kg ha^−1^ (average 835 ± 31.9), respectively. These values are within reported ranges (Selbie et al., 2015).

**Table 4b:** Average dung composition, and N and C loading rates for each experiment. DM = dry matter. Archived data sources: Bell et al. (2017); Cardenas et al. (2017); McGeough et al. (2017); Thorman et al., 2017a, Thorman et al., 2017b.

	Grazing season period	Total N loading (kg ha^−1^)	pH	DM (%)	Total N (g kg^−1^ DM)	NH_4_^+^-N (mg kg^−1^ DM)	NO_3_^−^-N (mg kg^−1^ DM)
Crichton	Early	1020	–	12.9	5.10	410	0.0
	Mid	680	–	11.5	3.40	260	0.0
	Late	720	–	10.6	3.60	230	0.0
Drayton	Early	840	7.6	18.9	22.2	5020	24.5
	Mid	736	7.6	36.2	10.2	3680	6.9
	Late	802	7.8	27.0	14.8	4443	0.0
Hillsborough	Early	980	6.9	14.5	4.90	500	0.0
	Mid	976	7.3	14.3	4.90	669	0.0
	Late	1008	7.7	14.3	5.00	683	0.0
North Wyke	Early	911	7.0	14.5	31.4	3035	0.1
	Mid	625	7.4	21.1	48.0	4310	21.6
	Late	771	7.5	20.5	18.8	2940	20.8
Pwllpeiran	Early	769	7.5	24.5	15.7	5095	25.2
	Mid	823	7.3	23.3	17.7	6497	7.9
	Late	866	7.5	24.1	18.0	5833	3.5

**Table 5:** Concentrations (g l^−1^) of purine derivatives in cattle urine used in the experiments. *Detection limit of the analytical approach. To convert from mg molecule l^−1^ to mg N l^−1^, multiply hippuric acid by 0.078138, allantoin by 0.354161, uric acid by 0.333115, and creatinine by 0.371287. Archived data sources: Bell et al. (2017); Cardenas et al. (2017); McGeough et al. (2017); Thorman et al., 2017a, Thorman et al., 2017b.

Site	Grazing season period	Hippuric acid	Allantoin	Uric acid	Creatinine
Crichton	Early	9.17	2.42	0.30	0.68
	Mid	1.57	1.70	0.45	1.29
	Late	7.69	3.89	0.41	0.77
Drayton	Early	<0.50*	2.83	0.48	0.58
	Mid	<0.50*	<0.40*	0.55	0.62
	Late	8.02	3.51	0.54	0.68
Hillsborough	Early	<0.50*	0.74	0.15	0.40
	Mid	<0.50*	<0.40*	0.06	<0.10*
	Late	<0.50*	<0.40*	0.12	<0.10*
North Wyke	Early	3.92	1.91	0.37	0.76
	Mid	<0.50*	<0.40*	0.40	0.52
	Late	4.86	<0.40*	0.35	0.52
Pwllpeiran	Early	5.13	0.84	0.36	0.81
	Mid	<0.50*	<0.40*	0.31	0.25
	Late	8.92	3.67	0.03	0.73

**Table 6:** Average cumulative N_2_O emissions and N_2_O EFs from the urine and dung treatments at each experimental site for each application. (Values in italics are standard errors of the mean). Archived data sources: Bell et al. (2017); Cardenas et al. (2017); McGeough et al. (2017); Thorman et al., 2017a, Thorman et al., 2017b. Within each site/timing experiment (rows), average total N_2_O emissions or N_2_O EFs between excretal N sources with different letters are significantly different (*p* < 0.05, *N* = 3).

		Cumulative emissions of N_2_O (kg N_2_O ha^−1^)					N_2_O EF (% of applied N)			
Site	Grazing season period	Control	Urine	Urine +DCD	Artificial urine	Dung	Urine	Urine +DCD	Artificial urine	Dung
Crichton	Early	0.96a *0.23*	1.92a *0.20*	1.25a *0.09*	0.93a *0.09*	2.15a *0.51*	0.20a *0.06*	0.06a *0.03*	−0.02a *0.13*	0.12a *0.03*
	Mid	0.61a *0.17*	5.18b *0.82*	5.07b *1.03*	5.29b *0.30*	2.00a *0.09*	1.09b *0.18*	1.05b *0.28*	1.10b *0.06*	0.20a *0.03*
	Late	0.79a *0.32*	2.21a *0.66*	1.79a *0.24*	1.49a *0.60*	1.55a *0.12*	0.33a *0.15*	0.23a *0.12*	0.16a *0.18*	0.11a *0.03*
Drayton	Early	0.18a *0.06*	2.02a *0.11*	1.35a *0.14*	1.86a *0.05*	0.85a *0.17*	0.34a *0.03*	0.21a *0.02*	0.34a *0.01*	0.08a *0.02*
	Mid	0.03a *0.09*	0.86a *0.08*	0.74a *0.07*	0.82a *0.08*	0.95a *0.05*	0.18a *0.00*	0.15a *0.00*	0.16a *0.01*	0.12a *0.01*
	Late	−0.03a *0.05*	7.68d *1.79*	4.73bc *1.35*	6.47 cd *0.42*	2.56b *0.35*	1.64c *0.37*	1.00b *0.28*	1.31bc *0.08*	0.32a *0.04*
Hillsborough	Early	0.36a *0.10*	4.78a *1.11*	1.46a *0.42*	10.87b *4.84*	1.98a *0.20*	1.02a *0.26*	0.25a *0.12*	2.06b *0.96*	0.17a *0.03*
	Mid	0.23a *0.04*	1.20a *0.15*	1.52a *0.44*	1.96a *0.61*	1.73a *0.45*	0.29a *0.05*	0.38a *0.14*	0.34a *0.13*	0.15a *0.04*
	Late	0.15a *0.05*	0.31a *0.07*	0.20a *0.04*	0.67a *0.11*	0.51a *0.15*	0.05a *0.03*	0.01a *0.00*	0.10a *0.03*	0.04a *0.01*
North Wyke	Early	1.26a *0.13*	13.26d *0.50*	5.54b *0.69*	11.06c *0.43*	2.50a *0.43*	2.96c *0.14*	1.09b *0.20*	2.23c *0.12*	0.14a *0.06*
	Mid	0.80a *0.07*	3.19b *0.51*	2.93b *0.50*	4.16b *0.89*	3.24b *0.52*	0.56b *0.11*	0.49ab *0.10*	0.70b *0.18*	0.39a *0.08*
	Late	0.03a *0.13*	0.52a *0.29*	0.59a *0.11*	0.34a *0.23*	0.82a *0.17*	0.11a *0.04*	0.12a *0.02*	0.07a *0.02*	0.10a *0.01*
Pwllpeiran	Early	0.49a *0.18*	3.45c *0.34*	1.59ab *0.31*	3.15c *0.23*	2.17bc *0.31*	0.52b *0.07*	0.19a *0.07*	0.54b *0.03*	0.22a *0.05*
	Mid	0.42a *0.04*	2.11b *0.13*	0.94ab *0.03*	1.81b *0.15*	2.13b *0.33*	0.30b *0.03*	0.09a *0.01*	0.28b *0.03*	0.21ab *0.04*
	Late	0.52a *0.07*	4.14b *0.39*	1.78a *0.13*	3.52b *0.36*	5.08c *0.81*	0.72c *0.09*	0.25a *0.02*	0.59bc *0.08*	0.53b *0.09*
Average of all sites and seasons							0.69 *0.20*	0.37 *0.09*	0.66 *0.18*	0.19 *0.03*

**Table 7:** Significance F-test probabilities for cumulative N_2_O emission and N_2_O EF, by timing of application, site, and timing of application × site interactions, from randomised block design ANOVA for each experiment.

		Treatments	Application time	Interaction
Crichton	Total N_2_O EF	<0.001 0.012	<0.001 <0.001	<0.001 0.019
Drayton	Total N_2_O EF	<0.001 <0.001	<0.001 <0.001	<0.001 0.002
Hillsborough	Total N_2_O EF	0.006 0.035	<0.001 0.002	0.014 0.042
North Wyke	Total N_2_O EF	<0.001 <0.001	<0.001 <0.001	<0.001 <0.001
Pwllpeiran	Total N_2_O EF	<0.001 <0.001	<0.001 <0.001	<0.001 0.093

### 3.2 Weather

Annual rainfall was greater than the 30-year mean in two (of the three) Crichton experiments, and all three experiments at Drayton, Hillsborough, North Wyke and Pwllpeiran. Average annual air temperature was similar to the 30-year mean at Crichton and Pwllpeiran, cooler at Hillsborough and North Wyke, and warmer at Drayton. However, it is more likely that the weather conditions immediately before urine and dung application, and within the first three months after application would have the most influence on N_2_O production and emission (see Table 3).

### 3.3 Nitrous oxide emissions

#### 3.3.1 Controls

Background (control) cumulative N_2_O emissions ranged from −0.03–1.26 kg N_2_O-N ha^−1^ for all sites and all experiments, with an average from the data in Table 6 of 0.49 kg N_2_O-N ha^−1^ (±0.10). From the meta-analysis, we find that across all seasons, the N_2_O emissions from the controls were significantly greater from the Crichton, North Wyke and Pwllpeiran sites compared to the Drayton site (*p* < 0.05). Within an individual site, emissions from controls also varied between seasons of application, particularly at the North Wyke site. There was no statistically significant relationship between the urine N_2_O EF and the cumulative annual N_2_O emission from the control plots (*p* > 0.05). Across all sites, N_2_O emissions from the control plots at the early grazing application timing were significantly greater than from the late-grazing application (*p* < 0.05). Regression modelling indicated that the key factors controlling the magnitude of the annual N_2_O fluxes from control plots were soil organic carbon content, clay content, bulk density, WFPS during the first 30d after application, and average annual temperature, with these factors accounting for ca. 56% of the variance in emissions. The resulting full regression equation was: Cumulative N_2_O flux (kg N ha^−1^) = 3.981–0.0846 SOC − 0.02220 initial WFPS + 0.01052 × 30d WFPS − 1.683 Bulk density − 0.01807 Clay content − 0.0408 × 365d average temperature.

#### 3.3.2 Urine

**Fig. 3:**
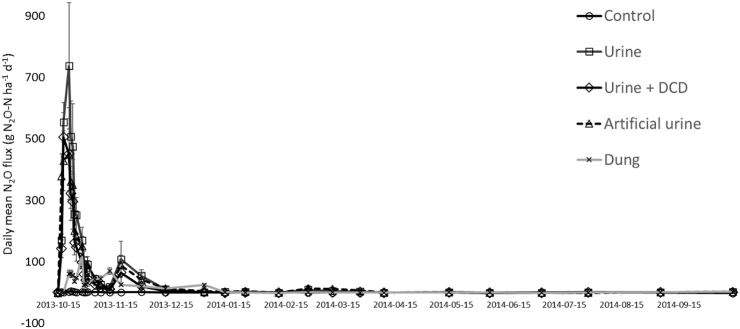
Daily mean N_2_O fluxes following urine and dung treatments at Drayton after a late-season application. (*N* = 3, vertical bars are standard error of the mean).

Examples of daily N_2_O fluxes are shown in Fig. 3 for the late-season urine, dung and control treatments at the Drayton site. These data show two distinct peaks in N_2_O fluxes, something observed in several of the experiments (e.g. Cardenas et al., 2016), suggesting the peaks in emission are associated either with different processes (e.g. denitrification of soil NO_3_ during the first peak as a result of the carbon addition in the urine, and nitrification of the urine NH_4_ source during the second peak), or different pools of N being the substrate for denitrification (e.g. the first peak associated with the urine-derived NH_4_, and the second peak associated with other more recalcitrant pools, e.g. N contained in purine derivatives). Further research using labelled urine N compounds would help reveal the underpinning processes and/or N sources responsible for the two peaks in emission.

**Fig. 4:**
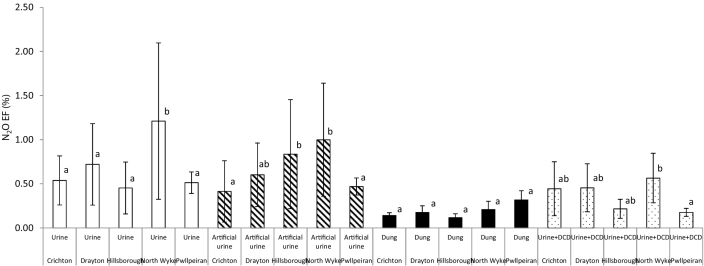
Average N_2_O EF (across three seasons of application) for each site, for urine and dung treatments. Within each urine/dung treatment, average N_2_O EFs (from meta-analysis) between sites with different letters are significantly different (*N* = 3, vertical lines are standard error of the mean).

**Fig. 5:**
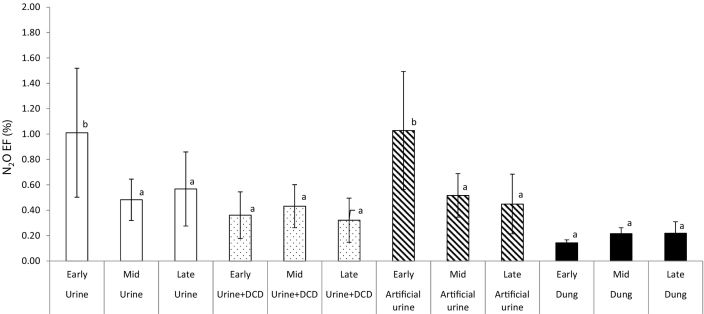
Effect of urine/dung treatment application timing (across all sites) on average N_2_O EF. Within each treatment, average N_2_O EFs (from meta-analysis) between timings of application with different letters are significantly different (*N* = 3, vertical lines are standard error of the mean).

The mean urine N_2_O EF was 0.69% (±0.20), ranging from 0.05–2.96 (Table 6). Across all seasons of application, the meta-analysis showed that the N_2_O EF was significantly greater from the North Wyke site than other sites (*p* < 0.05) (Fig. 4). Whilst across all sites, the N_2_O EF was significantly greater following an early-grazing application (*p* < 0.05) (Fig. 5). DCD reduced the N_2_O EF from urine in 13 of the 15 experiments, although this reduction was only significant in 5 of these experiments (Table 6). The average N_2_O EF for the urine + DCD treatment was 0.37% (±0.09) (Table 6). So, the use of DCD resulted in an average reduction in the N_2_O EF of 46%, although the range in efficacy was wide, i.e. from an increase in the N_2_O EF of 32% (mid-season application at Hillsborough) to a reduction of 75% (at the same site from the early-season application).

#### 3.3.3 Artificial urine

The mean artificial urine N_2_O EF was similar to that of the real urine, 0.66% (±0.18) (Table 6), and there was a good relationship between the N_2_O EFs for real and artificial urine (r^2^ = 0.77). Across all seasons, the meta-analysis showed that the N_2_O EF from the artificial urine was significantly greater at North Wyke and Hillsborough (*p* < 0.05) than the other sites (Fig. 4). Across all sites, the greatest N_2_O EF occurred following the early-grazing application (*p* < 0.05) (Fig. 5).

#### 3.3.4 Dung

The mean N_2_O EF for dung (from the meta-analysis) was 0.19% (±0.03), with a range of 0.04–0.53 (Table 6), which was significantly lower than for urine (*p* < 0.05). The meta-analysis showed there was no effect of site or season of application on the N_2_O EF from dung (*p* > 0.05) (Fig. 4, Fig. 5).

### 3.4 Factors affecting N_2_O fluxes from urine and dung

It is clear that there were significant (*p* < 0.05) effects of excretal N source and season of application at each site, as well as ‘treatment’ × ‘season’ interactions (Table 7).

#### 3.4.1 Urine

Multiple regression analysis showed that the factors that best explained cumulative N_2_O emissions from urine application mainly included urine composition and soil pH. The factors explaining 91.1% of the variance in cumulative N_2_O emissions from urine patches are shown via this equation: Cumulative N_2_O flux (kg N ha^−1^) = −61.94 + 38.50 urine creatinine content −0.0042 urine urea N content +0.003310 urine ammonium N content +0.002801 urine total nitrogen content +4.115 soil pH −1.036 urine hippuric acid content +4.340 urine pH −8.06 urine uric acid content. >75% of the variance in total N_2_O flux was explained by the urine total N, urea-N, ammonium-N, uric acid and creatinine content.

The full equation of factors explaining 91.1% of the urine N_2_O EF was; EF% = −15.9 + 8.776 urine creatinine content −0.0009595 urine urea N content −0.0007965 urine ammonium N content +1.014 soil pH +0.0005941 urine total nitrogen content −0.2563 urine hippuric acid content +1.116 urine pH −2.059 urine uric acid content. >75% of the variance in N_2_O EF was explained by the urine total N, urea-N, ammonium-N, uric acid and creatinine content.

#### 3.4.2 Dung

In contrast to urine, multiple regression showed that the factors that best explained cumulative N_2_O emissions from dung application included environmental and soil factors (as well as dung factors). The full equation, explaining 68.3% of the variance in cumulative N_2_O emissions from dung in this study was; Cumulative N_2_O flux (kg N ha^−1^) = 4.15–0.0579 initial %WFPS −0.308 365d average temperature −0.805 soil pH −0.0408 dung nitrate N content −0.00082 total nitrogen applied +1.053 soil organic carbon −10.50 soil dry bulk density +1.927 dung pH.

The full equation of factors explaining 66.5% of the dung N_2_O EF was; EF% = −0.295 + 0.0001187 dung ammonium N content +0.01784 30d %WFPS − 0.01473 dung nitrate N content − 0.002143 total nitrogen applied − 0.02343 30d average temperature + 0.1159 soil organic carbon +0.1747 dung total nitrogen content +0.0452 365d average temperature.

## 4 Discussion

Urine N_2_O EFs were significantly greater (average 0.69%) than the dung N_2_O EFs (average 0.19%), signifying the importance of the Nr content as a substrate for the soil processes, nitrification and denitrification, responsible for N_2_O production. Our urine and dung N_2_O EFs are similar to some of those measured by New Zealand researchers, summarised by Kelliher et al. (2014). In New Zealand, urine N_2_O EFs are categorised by livestock species and farming system (lowland, hill country low and high slope), and our results are more similar to the N_2_O EFs for the hill-country low slope dairy cattle urine (average of 0.84%) and dung (average of 0.20%). By contrast, Krol et al. (2016) reported larger average urine and dung N_2_O EFs for nine experiments conducted in Ireland of 1.18% (urine) and 0.39% (dung); EFs approximately double the values we have measured. In this series of experiments, Krol et al. (2016) applied urine at a higher N loading rate (average of 720 kg N ha^−1^) than in our study (average of 455 kg N ha^−1^). However, the greater N_2_O EF from the dung in the Irish study (0.39%) was despite using a lower N loading rate (average of 459 kg N ha^−1^) than in our study (835 kg N ha^−1^), suggesting that N loading was not the only factor resulting in the greater urine N_2_O EFs in these Irish experiments. Soil and environmental factors appeared to have been more conducive to N_2_O production and emission in this Irish study.

In our study, DCD reduced the urine N_2_O EFs by an average of 46%, although there was considerable variability in its efficacy to reduce N_2_O emissions (between sites and between seasons). In a related study, McGeough et al. (2016) took soil from these five UK grassland sites, and an additional four arable sites, and demonstrated that the efficacy of DCD to inhibit nitrification was controlled by the interaction between temperature, soil clay content and soil organic matter. Moreover, this study concluded that DCD was more effective in arable soils than in these grassland soils (McGeough et al., 2016). The average DCD N_2_O mitigation efficacy we measured (46%), and the range of efficacy that we measured are similar to other studies. For example, Selbie et al. (2014) showed that DCD increased the urine N_2_O EF by an average of +4% (a small increase) for urine applied at a loading rate of 500 kg N ha^−1^, but resulted in a 30% reduction for urine applied at 1000 kg N ha^−1^ (in New Zealand). Misselbrook et al. (2014) reported a greater efficacy of DCD to reduce the urine N_2_O EF, by 70% on a sandy clay loam in SW England. Recently, Minet et al. (2018) showed DCD, applied at 10 kg ha^−1^, could reduce the urine N_2_O EF by 34% (from 0.80% to 0.52%), but that DCD applied at 30 kg ha^−1^ reduced the urine N_2_O EF further, by 64%. Note: efficacy of DCD is often reported for cumulative emissions, with reported values being much higher than the efficacy of reducing the EF itself (e.g. Selbie et al., 2014). However, the efficacy of DCD to reduce N_2_O EFs is needed if national inventories are to be modified accordingly.

We found evidence of the effect of timing on N_2_O EFs, with larger EFs occurring following early-season urine application/deposition (Fig. 5). Krol et al. (2016) also explored the effect of season of urine application on N_2_O EFs from Irish grasslands, and showed that EFs varied seasonally, with the highest EFs in the autumn, and that emission were also dependent on soil type. Indeed, relationships between the magnitude of N_2_O EFs with “generic” season of deposition should be interpreted with caution, as soil and environmental conditions can vary markedly within a season. Hence, the importance of using statistical regression modelling to explore the key controls. Whilst there were insufficient data from our 15 experiments to be able to explore the relationships between cumulative N_2_O emissions, N_2_O EFs and climate/soil with certainty, the limited regression analysis showed that N_2_O emissions associated with urine were more related to urine composition than environmental and soil factors, whilst for dung which has a relatively low inorganic N content, N_2_O emissions were also controlled by soil and environmental factors. Krol et al. (2016) also used regression modelling to show the importance of rainfall and temperature before, and soil moisture deficit after, application of excretal deposition, on N_2_O emissions from nine experiments on Irish grasslands. We recognise the limitations of conducting regression analysis on such small data sets. However, there is potential to generate a much larger data set by combining data from studies where soils and climate are similar, and where similar protocols were followed, e.g. Krol et al. (2016), Minet et al. (2018), and data from some New Zealand experiments, to explore the controls of N_2_O emissions from urine and dung deposition, and generate improved EFs. Importantly, our unique dataset of daily N_2_O fluxes, cumulative emissions and emission factors, as well as soil mineral N and moisture data with weather, soil and site information have all been archived for future use by researchers (Bell et al., 2017; Cardenas et al., 2017; McGeough et al., 2017; Thorman et al., 2017a; Thorman et al., 2017b), and to allow integration with future datasets that become available.

To calculate a provisional excretal N_2_O EF, based on the data presented in this study, we assume a 60:40 split between the total N excreted in urine and dung (Webb and Misselbrook, 2004). We estimate a combined excretal N_2_O EF, based on our mean urine and dung N_2_O EFs data of 0.49%. These UK data have now been combined with the very few additional IPCC compliant UK experimental datasets (see Misselbrook et al., 2014) to generate a new country specific N_2_O EF of 0.44%. This is <25% of the IPCC (2006) default EF for cattle grazing excreta (EF_3_), and ca. 50% of the default EF for sheep grazing excreta. If we substitute this new pasture, range and paddock EF for both cattle and sheep into the IPCC, 2006 methodology for calculating the UK inventory, we estimate a reduction of 11.6 kt N_2_O (18% less N_2_O for UK agriculture for 2015) and for total UK agricultural GHG emissions, a reduction of 3.4 Mt. CO_2_e, or 7% for UK agriculture for 2015. This new EF is used in back-casting to 1990, and so has no bearing on meeting the UKs ambitious greenhouse gas mitigation target. However, a reduced GHG emission from agriculture means that a greater proportion of the emission can be “offset” by carbon sequestration, and suggests that e.g. land sparing strategies may be more realistic (Lamb et al., 2016). The lower country specific pasture, range and paddock EF_3_ also has implications for calculating carbon footprints of ruminant livestock products in the UK.

Clearly, this study focussed on cattle urine and dung where applications were made to lowland mineral soils, and where urine and dung were collected from cattle fed “lowland” diets. So, questions arise about a) extrapolating the N_2_O EF data to sheep; indeed the IPCC default sheep urine N_2_O EF (1%) is greater than the new combined cattle excreta N_2_O EF from our study, and b) extrapolating the new N_2_O EF data to beef and sheep grazing in the uplands, on much more organic and potentially acidic soils, and where weather and soil conditions as well as urine/dung composition may be very different.

## 5 Conclusions

This was the first co-ordinated study in the UK to generate data to develop a country specific grazing excreta N_2_O EF for cattle. Results confirmed that urine is the greatest source of N_2_O compared to dung, and that the nitrification inhibitor, DCD, offers the potential to reduce N_2_O emissions from urine patches, although its efficacy across the sites and seasons was variable. Understanding what controls this variability, and the development of cost effective delivery mechanisms need to be addressed if this technology is to be adopted. Importantly, the results of this study provide evidence that for the UK soil and climatic conditions, the N_2_O EF for grazing excreta for cattle is significantly lower (0.49%) than the IPCC default (2%) with implications for both government and the ruminant livestock industries as they seek to meet challenging greenhouse gas mitigation targets and greenhouse gas emission roadmaps, respectively. Further questions arise in terms of the validity of extrapolating these data from cattle to sheep grazing, and from mineral to organic soils.

## Data statement

Our unique datasets of daily N_2_O fluxes, cumulative emissions and emission factors, as well as soil mineral N and moisture data with weather, soil and site information from all 15 experiments have been archived for future use by researchers (see: Bell et al., 2017; Cardenas et al., 2017; McGeough et al., 2017; Thorman et al., 2017a; Thorman et al., 2017b), and to allow integration with future datasets that become available.
